# Retraction: MMP-9, uPAR and Cathepsin B Silencing Downregulate Integrins in Human Glioma Xenograft Cells *In Vitro* and *In Vivo* in Nude Mice

**DOI:** 10.1371/journal.pone.0328092

**Published:** 2025-07-11

**Authors:** The PLOS One Editors

After the publication of this article [1], concerns were raised about results presented in Figs 4 and 5. Specifically,

- The Fig 4A GAPDH panel of this article [1], appears to partially overlap with the Fig 5A GAPDH and the Fig 5E GAPDH panels of [2], despite being used to represent different experimental conditions.
- The Fig 5E 5310 Control panel appears to partially overlap with the Fig 5E 5310 SV-sh panel.

The authors did not respond to the journal’s communications.

In light of the above concerns, which call into question the reliability and integrity of the published image data and their associated quantified results, the *PLOS One* Editors retract this article.

All authors either did not respond directly or could not be reached.
